# Complete Genome Sequence of Rhodococcus qingshengii VT6, a Promising Degrader of Persistent Pollutants and Putative Biosurfactant-Producing Strain

**DOI:** 10.1128/mra.01179-21

**Published:** 2022-02-10

**Authors:** Yanina Delegan, Kirill Petrikov, Ekaterina Frantsuzova, Alexander Bogun, Vasili Travkin, Inna Solyanikova

**Affiliations:** a Federal Research Center “Pushchino Scientific Center for Biological Research of the Russian Academy of Sciences,” Pushchino, Moscow Region, Russian Federation; b The Federal State Budget Educational Institution of Higher Education Pushchino State Institute of Natural Science, Pushchino, Moscow Region, Russian Federation; c State Research Center for Applied Microbiology and Biotechnology, Obolensk, Russian Federation; d Belgorod State University, Belgorod, Russian Federation; University of Southern California

## Abstract

The strain Rhodococcus qingshengii VT6 is a promising degrader of persistent pollutants and a putative biosurfactant producer. The genome of the strain was sequenced completely. It consists of a 6,457,868-bp chromosome and 4 plasmids (pLP1, 501,672 bp; pLP2, 188,969 bp; pCP1, 100,387 bp; and pCP2, 132,858 bp).

## ANNOUNCEMENT

Rhodococci are known to be common representatives of soil microflora (1). They are known as metabolically versatile microorganisms with potential applications in bioremediation (2).

The Rhodococcus qingshengii strain VT6 (VKM Ac-2909D) was isolated from a forest soil sample (54°83′20″, 37°61'60″; Moscow Region, Russia Federation). The strain transforms hexadecane and trinitrotoluene (3) and produces surface-active compounds. Surface tension (ST) was measured by the du Noüy method (4) using the tensiometer K6 (Kruss, Germany) at the temperature of 25°C. Cultivation of the strain in liquid Evans (5) medium (reference solution, ST 77 mN·m^−1^) with hexadecane 2% (vol/vol) at 25°C for 5 days resulted in the reduction of ST to 36 mN·m^−1^, which may indicate the synthesis of surfactants.

For long-term storage, the strain was kept in glycerol (40%) at −70°С. For short-term maintenance, the strain was cultured on LB (6) agar plates at 27°C.

Genomic DNA was isolated from a fresh biomass of *Rhodococcus* VT6 grown on LB agar using a DNeasy kit (catalog [cat.] no 69506; Qiagen). The 16S rRNA gene sequencing was performed as described in reference 3. The analysis of the sequencing results showed that the strain is related to Rhodococcus erythropolis or *R. qingshengii*. The closest relative of the VT6 strain is *R. qingshengii* TG-1 (GenBank accession no. CP077417.1).

Sequencing was performed in Federal Research Center “Pushchino Scientific Center for Biological Research, RAS” using a MinION sequencer with the flow cell R9.4.1 (Oxford Nanopore Technologies). A library was prepared with a ligation kit (SQK-LSK109). Guppy 3.2.4 was used for base calling, which yielded a total of 1,306.8 Mb distributed in 130,800 reads with a Q of >10 (*N*_50_ is 17,156 bp).

The same DNA sample was sequenced with an Illumina NovaSeq6000 instrument using an S2 reagent kit (cat. no. 20012861) in BioSpark (Troitsk, Russia). A paired-end library was prepared with the HyperPlus kit (Kapa Biosystems). We obtained 37,607,400 paired-end reads of <101 bp. Quality control was performed using FastQC (7) and NanoPack (8). The Illumina and Nanopore reads were used for hybrid assembly with SPAdes 3.15.0 (9). The Nanopore reads were assembled into 5 contigs using Flye 2.9 (10). Next, SPAdes contigs were combined into replicons using Snapgene 6.0 with Flye data as the reference. The Illumina reads were used to correct Nanopore errors using Bowtie 2 2.4.4 (11) and Pilon 1.24 (12). Default parameters were used for all software unless otherwise specified.

The *R. qingshengii* VT6 genome consists of a 6,457,868-bp chromosome (GC content, 62.41%) and 4 plasmids. The plasmids pLP1 (501,672 bp; GC content, 61.50%) and pLP2 (188,969 bp; GC content, 61.47%) are linear, pCP1 (100,387 bp; GC content, 61.88%) and pCP2 (132,858 bp; GC content, 63.24%) are circular. All plasmids also were visualized using pulsed-field electrophoresis. Chromosome and plasmid circularization were specified by end overlapping and using Tablet 1.21.02.08 (13). The ends of linear plasmids were verified by amplification (see supplemental data online at FigShare [10.6084/m9.figshare.18758252]) and subsequent sequencing.

To identify the strain to species, we used average nucleotide identity (ANI) value (https://www.ezbiocloud.net/tools/ani [14]) and digital DNA-DNA hybridization (DDH) (https://ggdc.dsmz.de/ggdc.php [15]). The ANI value and DDH with the type strain R. erythropolis NBRC15567 are 95.38% and 75.60%, respectively, and with the type strain *R. qingshengii* JCM15477 are 98.45% and 77.30%, respectively. So, we identify the strain VT6 as *R. qingshengii*.

The genome of strain VT6 was annotated using NCBI PGAP 4.6 (16) and Prokka 1.14.6 (17). The strain bears a number of catabolic genes for alkane degradation. We found a set of trehalose biosynthesis genes. Trehalose is one of the main components of glycolipid biosurfactants. The VT6 strain is a potential producer of biosurfactants.

### Data availability.

This genome project has been deposited at GenBank under BioSample SAMN23500510, BioProject PRJNA784759, GenBank accession numbers CP088906 to CP088910, and SRA accession numbers SRX13270910 for Illumina and SRX13270985 for Oxford Nanopore data.
